# lncRNA-ZFAS1 promotes the progression of endometrial carcinoma by targeting miR-34b to regulate VEGFA expression

**DOI:** 10.1515/med-2021-0362

**Published:** 2021-10-04

**Authors:** Hongli Zhu, Qihui Cheng, Hong Cai

**Affiliations:** Department of Gynecology and Obstetrics, Affiliated Hangzhou First People’s Hospital of Zhejiang University, No. 1 Xueshi Road, Shangcheng District, Hangzhou City, Zhejiang Province, 310010, China

**Keywords:** ZFAS1, miR-34b, VEGFA, endometrial carcinoma, proliferation

## Abstract

Zinc finger nuclear transcription factor, X-box binding 1-type containing 1 antisense RNA 1 (ZFAS1) functions as an oncogenic long noncoding RNA (lncRNA) to promote proliferation and metastasis of endometrial carcinoma cell; however, the underlying mechanism has not been fully understood. First, RT-qPCR analysis of endometrial carcinoma tissues and cells showed that ZFAS1 was enriched in endometrial carcinoma tissues and cells. miR-34b was reduced in endometrial carcinoma and suggested negative correlation with ZFAS1 in endometrial carcinoma. Second, functional assays demonstrated that siRNA-mediated silence of ZFAS1 suppressed endometrial carcinoma cell proliferation and metastasis. Third, ZFAS1 bind to miR-34b and negatively regulate expression of miR-34b in endometrial carcinoma cells. miR-34b also bind to and negatively regulate expression of vascular endothelial growth factor A (VEGFA) in endometrial carcinoma cells. Lastly, knockdown of miR-34b counteracted with the suppressive effects of ZFAS1 silence on endometrial carcinoma cell proliferation and metastasis. In conclusion, lncRNA ZFAS1 functioned as an oncogene to promote endometrial carcinoma cell proliferation and metastasis through miR-34b/VEGFA axis.

## 1 Introduction

Endometrial cancer is a common epithelial malignancy of the female reproductive system, with the fourth highest incidence among female malignancies and the second leading cause of death from gynecological cancer worldwide [1]. Standard treatment, such as surgical resection, has improved the overall survival rate of patients at early stage of endometrial cancer, while has little effect on the survival rate of patients at advanced stage [2]. The 5-year overall survival rate of patients with advanced endometrial cancer is less than 20% [3]. Therefore, investigation of therapeutic targets for endometrial cancer is of great importance to improve the survival rate.

In recent years, noncoding RNAs, including long noncoding RNA (lncRNAs) and microRNAs (miRNAs), have been shown to be key regulators of cancer, including endometrial cancer [4]. Meanwhile, lncRNAs are reported to be involved in almost all cancer metastasis processes, including proliferation, invasion, and metastasis, through transcriptional or post-transcriptional regulation as well as scaffolds for lncRNA, miRNA, and protein interactions [5]. lncRNA Zinc finger nuclear transcription factor, X-box binding 1-type containing 1 antisense RNA 1 (ZFAS1) was reported to be a prognostic factor in glioma [6] and multiple human cancers [7], and participated in progression of gastric cancer [8], nasopharyngeal carcinoma [9], hepatocellular carcinoma [10], and head and neck squamous cell carcinomas [11]. Downregulation of ZFAS1 repressed thyroid carcinoma progression [12]. Recent study showed that ZFAS1 promoted endometrial carcinoma cell proliferation and epithelial–mesenchymal transition [13]. However, the underlying mechanism involved in ZFAS1-mediated endometrial carcinoma progression has not been fully understood.

lncRNAs function as competing endogenous RNA to mediate miRNA–mRNA signature and participate in progression of endometrial carcinoma [14]. ZFAS1 has been reported to sponge miR-1271-5p to promote lung adenocarcinoma progression [15], and regulate ovarian cancer metastasis through sponging miR-548e [16]. miR-34b was predicted as binding target of ZFAS1 via StarBase website [17]. miR-34b was reported to be a tumor suppressor of endometrial serous adenocarcinoma [18], and miR-34b suppressed endometrial carcinoma cell growth and metastasis [19]. Therefore, we hypothesized that ZFAS1 might promote endometrial carcinoma progression through sponging of miR-34b.

This study first evaluated the expression level of ZFAS1 in endometrial carcinoma tissues and cells and then assessed the functional role of ZFAS1 on endometrial carcinoma cell growth and metastasis. The downstream miRNA–mRNA target involved in ZFAS1-mediated endometrial carcinoma progression was determined in this study.

## 2 Methods

### 2.1 Collection of tissue specimen

Sixty pair of tumor and adjacent tissues were collected from patients with endometrial carcinoma via surgical resection. The patients with written informed consents were recruited at Affiliated Hangzhou First People’s Hospital of Zhejiang University, and the experiment was approved by Affiliated Hangzhou First People’s Hospital of Zhejiang University and in accordance with 1964 Helsinki Declaration.

### 2.2 Cell culture and transfection

Immortalized stromal cell line (hEM15A) and endometrial carcinoma cell lines (HEC-1B, Ishikawa, KLE, and RL-952) were purchased from ATCC (Manassas, VA, USA). DMEM medium (HyClone, Logan, UT, USA) containing 10% of fetal bovine serum (Gibco BRL, Gaithersburg, MD, USA) was used to culture the cells at 37°C in a humidified incubator. The siRNAs targeting ZFAS1 (si-ZFAS1#1 or #2), si- vascular endothelial growth factor A (VEGFA), pcDNA-ZFAS1, miR-34b mimic, and inhibitor, as well as the negative controls (si-NC, pc-DNA, NC-mimic, and inh NC), were synthesized by GenePharma (Suzhou, China). HEC-1B was transfected with siRNAs, pc-DNAs, mimic, or inhibitor via Lipofectamine 2000 (Thermo Fisher Scientific, Waltham, MA, USA).

### 2.3 Cell viability and proliferation

HEC-1B and Ishikawa were plated in a 96-well plate for 24 h and then conducted with indicated transfections. Cells in each well were incubated with 10 μL CCK8 solution (Sigma-Aldrich, St. Louis, MO, USA) at indicated time post transfection (0, 24, 48, 72 h) for 2 h. Absorbance at 450 nm was measured by a spectrometer (Thermo Fisher Scientific). HEC-1B and Ishikawa were plated in a 6-well plate for 24 h and then conducted with indicated transfections. Two weeks later, cells in each well were fixed in 10% of formaldehyde and stained with 0.4% of crystal violet (Sigma-Aldrich). The colonies were measured under a light microscope (Olympus Corp. Tokyo, Japan).

### 2.4 Cell migration and invasion

HEC-1B and Ishikawa were plated in a 6-well plate for 24 h and then conducted with indicated transfections for another two days. A pipette tip was used to generate wound scratch in each well. Twenty-four hours later, the wound width was calculated under microscope (Olympus Corp.). Transfected HEC-1B and Ishikawa were suspended in 200 μL serum-free DMEM medium and then seeded into the upper chamber of Transwell insert chamber (Corning, Tewksbury, MA, USA) with matrigel coating (BD Bioscience, San Jose, CA, USA). The lower chamber was filled with 600 µL of DMEM containing 10% of fetal bovine serum. Twenty-four hours later, the invasive cells in the lower chamber were fixed in 4% of paraformaldehyde and stained with 1% of crystal violet. Cells were measured under microscope (Olympus Corp.).

### 2.5 Cell apoptosis and luciferase reporter assays

HEC-1B and Ishikawa were harvested and then suspended in binding buffer from ApoDETECT Annexin V-FITC Kit (Thermo Fisher Scientific Inc). The cells were analyzed by FACS flow cytometer (Attune, Life Technologies, Darmstadt, Germany) followed by incubation with Annexin V-FITC and PI (Thermo Fisher Scientific Inc). The wildtype sequences of ZFAS1 or VEGFA containing the binding sites of miR-34b were subcloned into the pmirGLO vector (Promega, Madison, WI, USA). The mutant sequences with mutations at the binding sites of miR-34b were also subcloned into the pmirGLO vector. HEC-1B was cotransfected with the pmirGLO vectors and miR-34b mimic or NC mimic for two days. The luciferase activities were determined by luciferase reporter assay kit (Promega).

### 2.6 RNA immunoprecipitation

HEC-1B was lysed in RIPA lysis buffer (Millipore, Bedford, MA, USA), and the cell extracts (100 μL) were incubated with magnetic beads conjugated to anti-Ago2 or anti-IgG antibodies. Proteinase K was used to digest the proteins, and the immunoprecipitated RNAs were isolated and conducted with qRT-PCR analysis.

### 2.7 qRT-PCR

RNAs were isolated from endometrial carcinoma tissues or cells by Trizol (Thermo Fisher Scientific), and miRcute miRNA isolation kit (Tiangen, Beijing, China) was used to extract miRNAs. RNAs and miRNAs were then converted into cDNAs by MMLV reverse transcriptase (Promega). The fold changes of ZFAS1, miR-34b and VEGFA expression compared to U6 or GAPDH were determined by SYBR Green Master (TaKaRa, Dalian, China) with primer sequences as listed in Table 1.

**Table 1:** Primer

ID	Sequence (5′–3′)
GAPDH F	AGGTCGGTGTGAACGGATTTG
GAPDH R	TGTAGACCATGTAGTTGAGGTC
U6 F	CTCGCTTCGGCAGCACA
U6 R	AACGCTTCACGAATTTGCGT
miR-34b F	CCAGGACCAGAGGAAACCT
miR-34b R	GCTAGCCTCTGGATTTGA
ZFAS1 F	ACGTGCAGACATCTACAACCT
ZFAS1 R	TACTTCCAACACCCGCAT
VEGFA F	TTGCCTTGCTGCTCTACCT
VEGFA R	GATGGCAGTAGCTGCGCTG

### 2.8 Western blot

Endometrial carcinoma cells were lysed in RIPA Lysis and Extraction Buffer (Thermo Fisher Scientific), and the protein concentration of the cellular lysates was determined by acid protein kit (Thermo Fisher Scientific). The lysates were separated by SDS-PAGE and then electro-transferred onto PVDF membrane (Millipore, Bedford, MA, USA). The membranes were blocked in 5% of BSA, and probed overnight with the primary antibodies: anti-VEGFA (1:2,000, Cell Signaling, Beverly, MA, USA) and anti-β-actin (1:3,000, Cell Signaling). Following incubation with horseradish peroxidase-labeled secondary antibody (1:5,000; Cell Signaling), the immunoreactivities were detected by enhanced chemiluminescence (KeyGen, Nanjin, China).

### 2.9 Statistical analysis

Data were expressed as mean value ± SEM, and analyzed with one-way analysis of variance or student’s *t* test under GraphPad Prism software. The *p* value <0.05 was considered as statistically significant.

**Ethics approval:** Ethical approval was obtained from the Ethics Committee of Affiliated Hangzhou First People’s Hospital of Zhejiang University.**Informed consent:** Written informed consent was obtained from a legally authorized representative(s) for anonymized patient information to be published in this article.

## 3 Results

### 3.1 Elevated ZFAS1 in endometrial carcinoma

**Figure 1:**
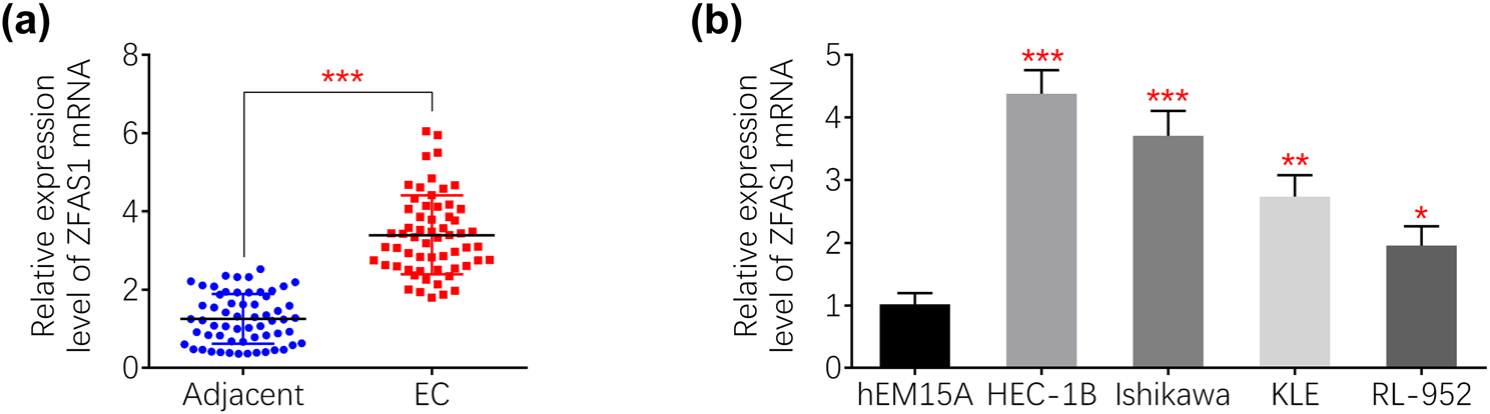
Elevated ZFAS1 in endometrial carcinoma. (a) ZFAS1 was enhanced in the endometrial carcinoma tissues compared to adjacent tissues. (b) ZFAS1 was enhanced in the endometrial carcinoma cell lines (HEC-1B, Ishikawa and KLE, RL-952) compared to hEM15A. *p < 0.05, **p < 0.01 and ***p < 0.0001.

To investigate the relation between ZFAS1 and endometrial carcinoma, expression level of ZFAS1 in endometrial carcinoma tissues and cells were evaluated by qRT-PCR. As shown in Figure 1a, ZFAS1 was enhanced in the endometrial carcinoma tissues compared to adjacent tissues. Similarly, upregulation of ZFAS1 in endometrial carcinoma cell lines (HEC-1B, Ishikawa, KLE, and RL-952) was also verified by qRT-PCR (Figure 1b).

### 3.2 Silence of ZFAS1 suppressed endometrial carcinoma cell growth and metastasis

**Figure 2:**
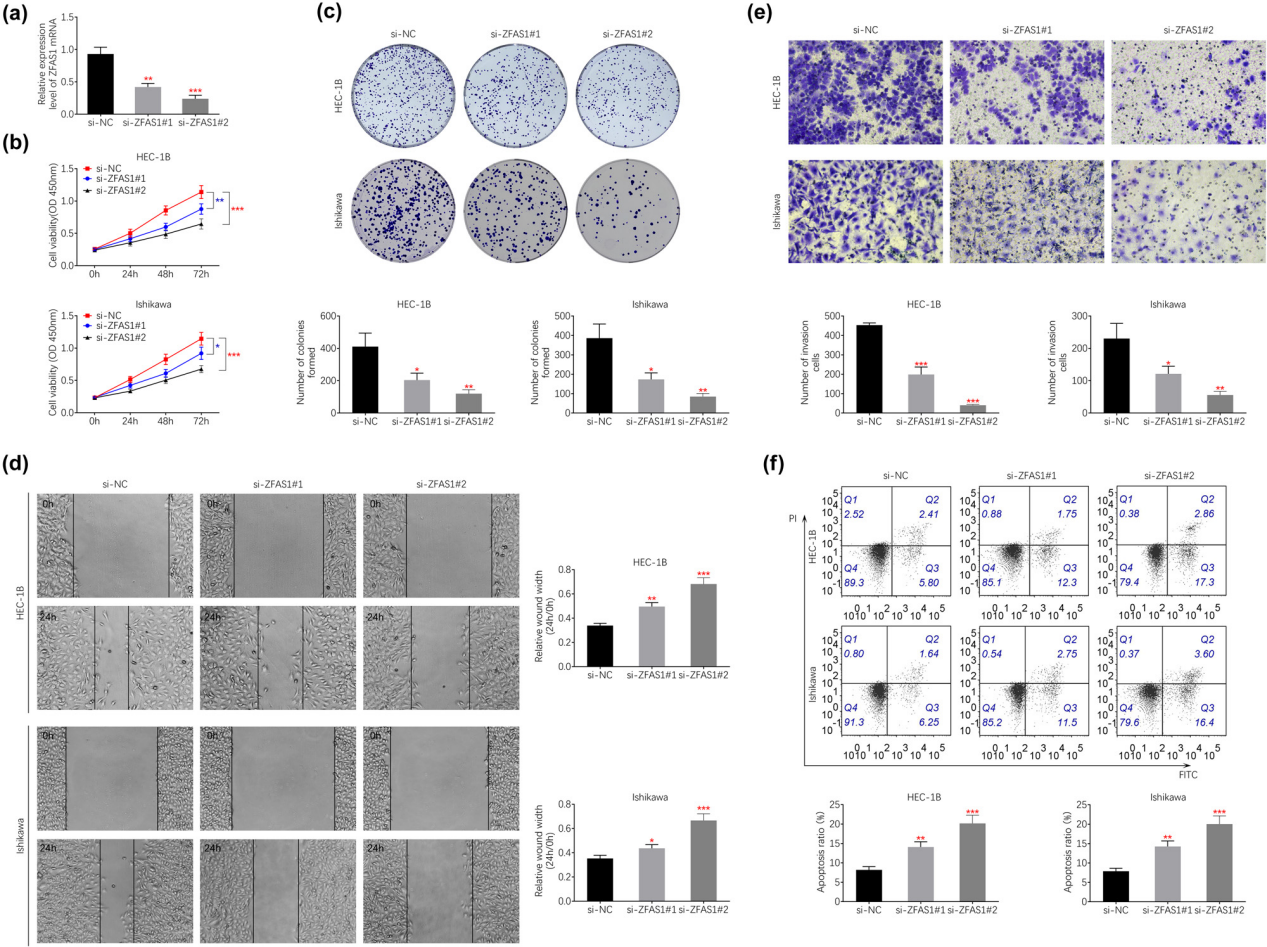
Silence of ZFAS1 suppressed endometrial carcinoma cell growth and metastasis. (a) Transfection with si-ZFAS1#1 or #2 decreased expression of ZFAS1 in HEC-1B. (b) Transfection with si-ZFAS1#1 and #2 decreased cell viability of HEC-1B and Ishikawa. (c) Transfection with si-ZFAS1#1 and #2 suppressed cell proliferation of HEC-1B and Ishikawa. (d) Transfection with si-ZFAS1#1 and #2 suppressed cell migration of HEC-1B and Ishikawa. (e) Transfection with si-ZFAS1#1 and #2 suppressed cell invasion of HEC-1B and Ishikawa. (f) Transfection with si-ZFAS1#1 and #2 promoted cell apoptosis of HEC-1B and Ishikawa.**p < 0.01 and ***p < 0.0001.

Loss-of-functional assays were then applied to detect the role of ZFAS1 on endometrial carcinoma progression. siRNAs-mediated knockdown of ZFAS1 was verified by qRT-PCR (Figure 2a). si-ZFAS1#2 showed lower expression of ZFAS1 than si-ZFAS1#1 (Figure 2a). Transfection with si-ZFAS1#1 and #2 decreased cell proliferation of HEC-1B and Ishikawa (Figure 2b and c). Cell migration (Figure 2d) and invasion (Figure 2e) were repressed by silence of ZFAS1. Moreover, transfection with si-ZFAS1#1 and #2 promoted cell apoptosis of HEC-1B and Ishikawa (Figure 2f). These results suggested the anti-proliferative and anti-invasive capacities of ZFAS1 silence on endometrial carcinoma progression.

### 3.3 ZFAS1 bind to miR-34b

**Figure 3:**
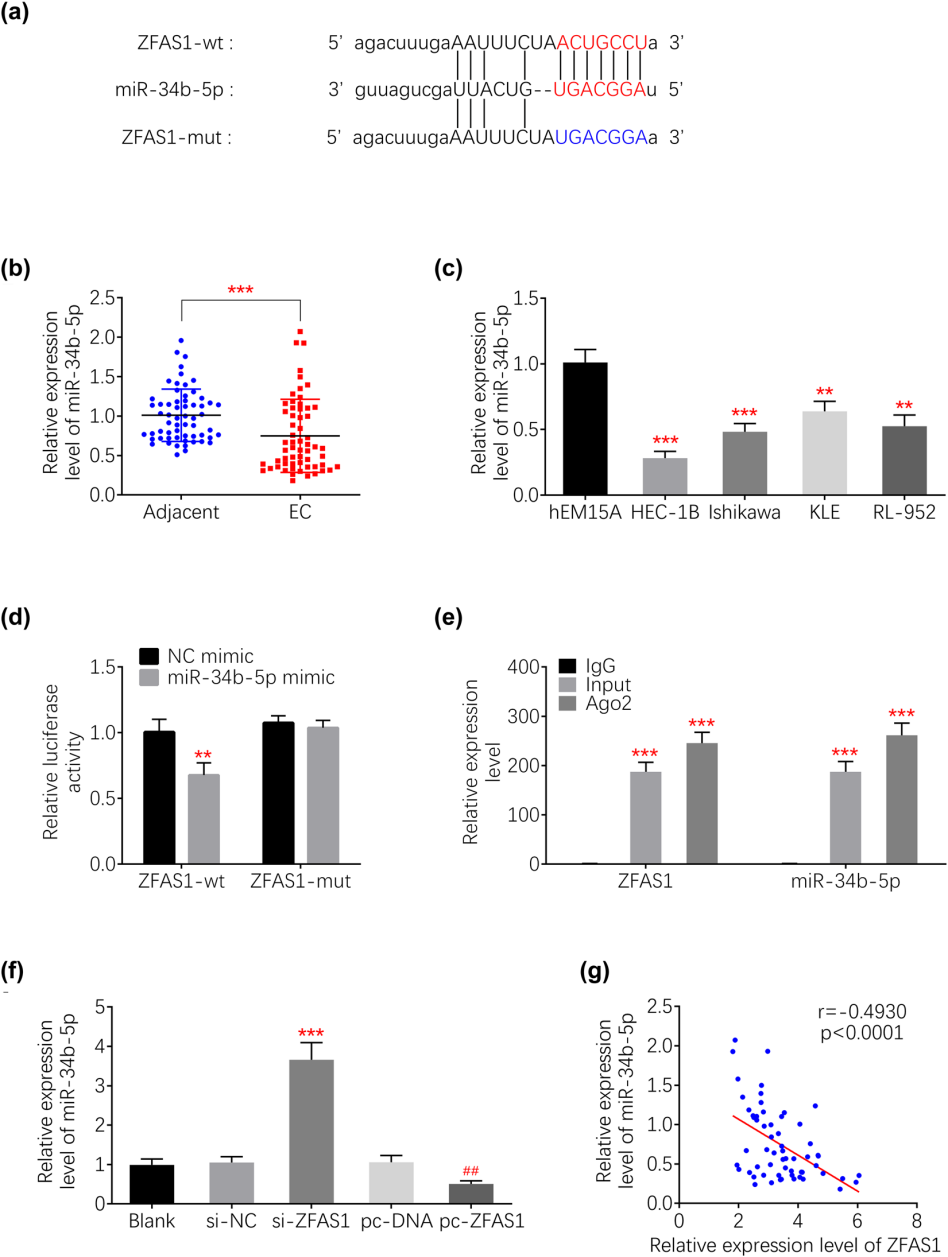
ZFAS1 bind to miR-34b. (a) Potential binding site between ZFAS1 and miR-34b. (b) miR-34b was reduced in the endometrial carcinoma tissues compared to adjacent tissues. (c) miR-34b was reduced in the endometrial carcinoma cell lines (HEC-1B, Ishikawa, KLE and RL-952) compared to hEM15A. (d) miR-34b mimic decreased activity of wild type pmirGLO-ZFAS1, while it had no significant effect on mutant pmirGLO-ZFAS1. (e) Expression of miR-34b was decreased in HEC-1B transfected with si-ZFAS1#2, while increased by pcDNA-mediated over-expression of ZFAS1. (f) Negative correlation between ZFAS1 and miR-34b in endometrial carcinoma tissues. **, ^##^ *p* < 0.01 and ***p < 0.0001.

Potential sponging miRNA of ZFAS1 was predicted as miR-34b via lncBase (Figure 3a). Expression level of miR-34b was reduced in endometrial carcinoma tissues (Figure 3b) and cells (Figure 3c). Luciferase activity assay confirmed the binding ability between miR-34b and ZFAS1 by demonstrating that miR-34b mimic decreased the activity of wildtype pmirGLO-ZFAS1, while it had no significant effect on mutant pmirGLO-ZFAS1 (Figure 3d). RNA immunoprecipitation showed that miR-34b and ZFAS1 were enriched in magnetic beads conjugated with anti-AGO2 (Figure 3e), further confirming that ZFAS1 bind to miR-34b. Moreover, expression of miR-34b decreased in HEC-1B transfected with si-ZFAS1#2, while increased by pcDNA-mediated over-expression of ZFAS1 (Figure 3f). The negative correlation between ZFAS1 and miR-34b in endometrial carcinoma tissues (Figure 3g) suggests that ZFAS1 might regulate endometrial carcinoma progression through sponging of miR-34b.

### 3.4 miR-34b bind to VEGFA

**Figure 4:**
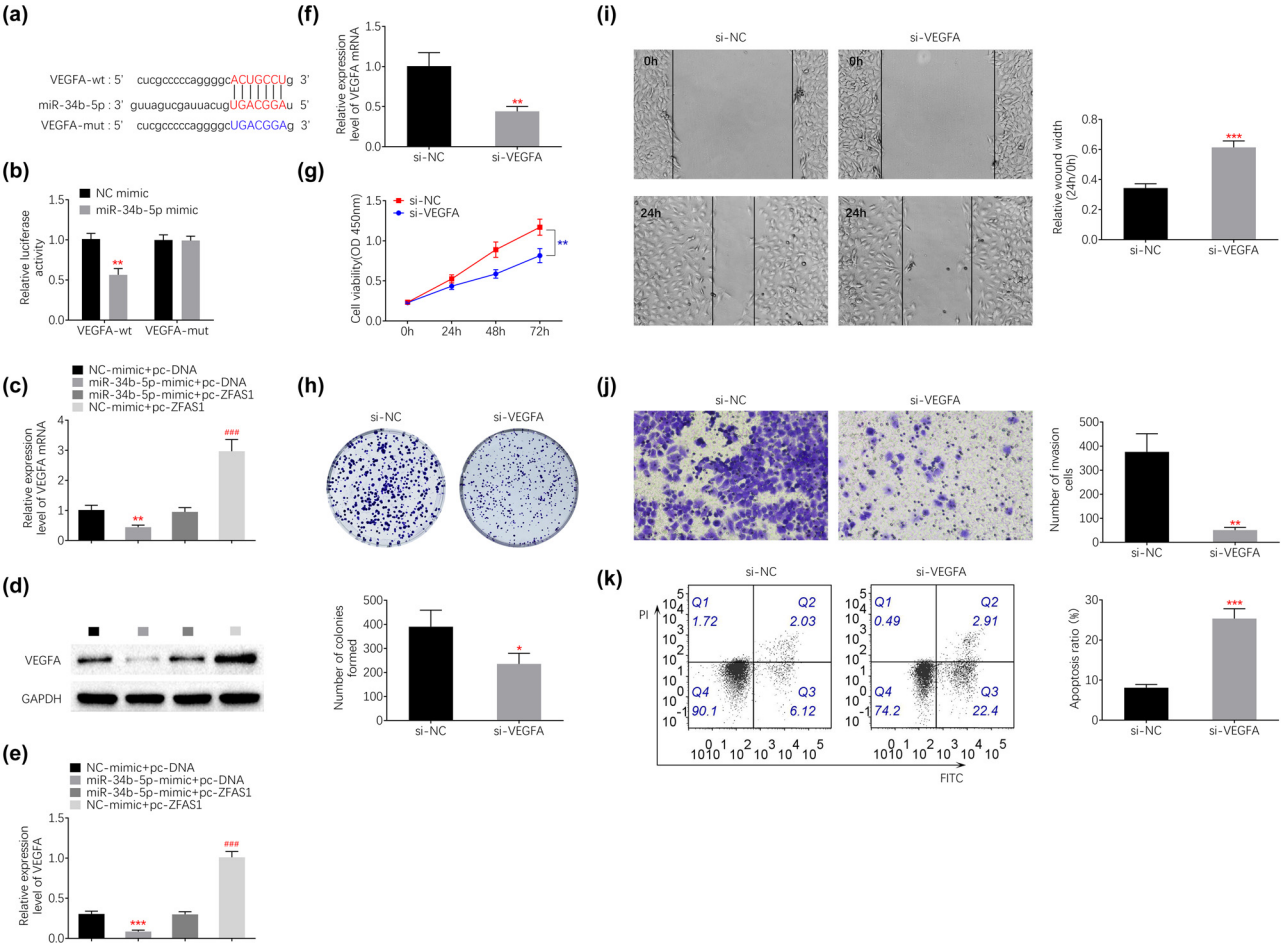
miR-34b bind to VEGFA. (a) Potential binding site between miR-34b and VEGFA. (b) miR-34b mimic decreased activity of wild type pmirGLO-VEGFA, while it had no significant effect on mutant pmirGLO-VEGFA. (c) mRNA expression of VEGFA was decreased in HEC-1B transfected with miR-34b mimic, while increased by pcDNA-ZFAS1. Over-expression of ZFAS1 attenuated miR-34b mimic-induced decrease in VEGFA. (d) Protein expression of VEGFA was decreased in HEC-1B transfected with miR-34b mimic, while increased by pcDNA-ZFAS1. Over-expression of ZFAS1 attenuated miR-34b mimic-induced decrease in VEGFA. (e) Protein expression of VEGFA was decreased in HEC-1B transfected with miR-34b mimic, while increased by pcDNA-ZFAS1. Over-expression of ZFAS1 attenuated miR-34b mimic-induced decrease in VEGFA. (f) Transfection with si-VEGFA reduced mRNA expression of VEGFA. (g) Transfection with si-VEGFA decreased cell viability of HEC-1B. (h) Transfection with si-VEGFA decreased cell proliferation of HEC-1B. (i) Transfection with si-VEGFA decreased cell migration of HEC-1B. (j) Transfection with si-VEGFA decreased cell invasion of HEC-1B. (k) Transfection with si-VEGFA promoted cell apoptosis of HEC-1B. **p < 0.01 and ***, ^###^ *p* < 0.0001.

Potential binding mRNA of miR-34b was predicted as VEGFA via starBase (Figure 4a). Luciferase activity assay demonstrated that miR-34b mimic decreased the activity of wildtype pmirGLO-VEGFA, while it had no significant effect on mutant pmirGLO-VEGFA (Figure 4b), showing that miR-34b bind to VEGFA. Moreover, mRNA (Figure 4c) and protein (Figure 4d and e) expression of VEGFA were decreased in HEC-1B transfected with miR-34b mimic, while increased by ZFAS1 over-expression (Figure 4c–e). pcDNA-mediated over-expression of ZFAS1 attenuated miR-43b mimic-induced decrease in mRNA (Figure 4c) and protein (Figure 4d and e) expression of VEGFA, indicating that ZFAS1 might sponge miR-34b to increase VEGFA in endometrial carcinoma. HEC-1B was transfected with si-VEGFA (Figure 4f). Knockdown of VEGFA decreased cell proliferation (Figure 4g and h), suppressed cell migration (Figure 4i) and invasion (Figure 4j), and promoted the cell apoptosis (Figure 4k) of HEC-1B.

### 3.5 Silence of ZFAS1 suppressed endometrial carcinoma cell growth and metastasis through increase in miR-34b

**Figure 5:**
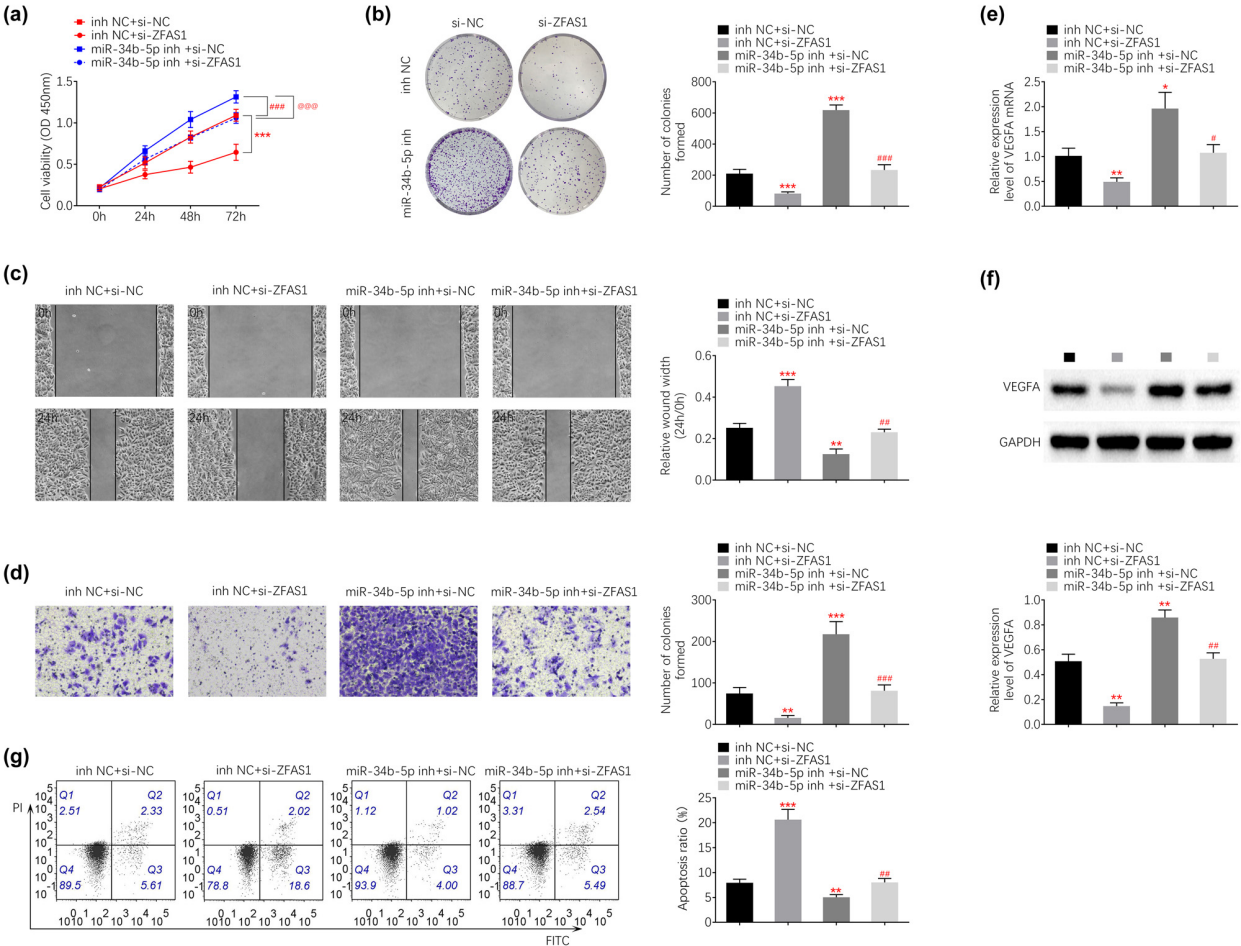
Silence of ZFAS1 suppressed endometrial carcinoma cell growth and metastasis through increase in miR-34b. (a) Transfection with miR-34b inhibitor increased cell viability of HEC-1B, and attenuated ZFAS1 silence-induced decrease in cell viability. (b) Transfection with miR-34b inhibitor increased cell proliferation of HEC-1B, and attenuated ZFAS1 silence-induced decrease in cell proliferation. (c) Transfection with miR-34b inhibitor increased cell migration of HEC-1B, and attenuated ZFAS1 silence-induced decrease in cell migration. (d) Transfection with miR-34b inhibitor increased cell invasion of HEC-1B, and attenuated ZFAS1 silence-induced decrease in cell invasion. (e) Transfection with miR-34b inhibitor increased mRNA expression of VEGFA, and attenuated ZFAS1 silence-induced decrease in VEGFA. (f) Transfection with miR-34b inhibitor increased protein expression of VEGFA, and attenuated ZFAS1 silence-induced decrease in VEGFA. (g) Transfection with miR-34b inhibitor decreased cell apoptosis of HEC-1B, and attenuated ZFAS1 silence-induced increase in cell apoptosis. *, ^#^ *p* < 0.05, **, ^##^ *p* < 0.01 and ***, ^###^ *p* < 0.0001.

To investigate the role of ZFAS1/miR-34b axis on endometrial carcinoma progression, HEC-1B was cotransfected with si-ZFAS1#2 and miR-34b inhibitor. Transfection with miR-34b inhibitor increased cell viability of HEC-1B (Figure 5a), and attenuated ZFAS1 silence-induced decrease in cell viability (Figure 5a). Cell proliferation (Figure 5b), migration (Figure 5c), and invasion (Figure 5d) were promoted by knockdown of miR-34b. Knockdown of miR-34b counteracted with the suppressive effects of ZFAS1 silence on endometrial carcinoma cell proliferation (Figure 5b), migration (Figure 5c), and invasion (Figure 5d). Moreover, silence of ZFAS1-induced decrease in mRNA (Figure 5e) and protein (Figure 5f) expression of VEGFA were reversed by interference of miR-34b. The increased cell apoptosis of HEC-1B induced by silence of ZFAS1 was restored by inhibition of miR-34b (Figure 5g), revealing that silence of ZFAS1 suppressed endometrial carcinoma cell growth and metastasis through increase in miR-34b and decrease in VEGFA.

## 4 Discussion

lncRNAs have gained great attention due to the roles in carcinogenesis and cancer progression of endometrial carcinoma [20]. Dysregulated lncRNAs in endometrial carcinoma might promote or suppress the cancer progression, as well as predict poor prognosis [21]. Previous study has shown that upregulation of ZFAS1 in endometrial carcinoma predicted poor prognosis of the patients, and *in vitro* loss-of-functional assays validated the oncogenic role of ZFAS1 on endometrial carcinoma [13]. The mechanism was then verified in this study.

In line with the previous study [13], results in this study also showed that ZFAS1 was enhanced in endometrial carcinoma tissues and cells, and knockdown of ZFAS1 contributed to the suppression of endometrial carcinoma cell growth and metastasis. Moreover, miR-34b was verified as the sponging miRNA of ZFAS1 in endometrial carcinoma. Three homologous miRNAs, including miR-34a, miR-34b, and miR-34c, in the miR-34 family, have been shown to be dysregulated in cancer tissues, and participate in the occurrence and development of tumors [22]. miR-34a [23] and miR-34b [18] suppressed endometrial carcinoma progression, and miR-34b enhanced paclitaxel-induced cytotoxity in endometrial carcinoma cells [24]. Knockdown of miR-34b in this study contributed to endometrial carcinoma cell growth and metastasis, and attenuated ZFAS1 silence-induced suppression of endometrial carcinoma cell growth and metastasis. Therefore, ZFAS1 might promote endometrial carcinoma progression through sponging of miR-34b.

Results in this study showed that miR-34b bind to VEGFA in endometrial carcinoma cells. VEGFA was reported to be involved in miR-34b-5p-mediated angiogenesis [25] and cell growth [26] of thyroid carcinoma. Reduction of VEGFA by over-expression of miR-34b suppressed thyroid carcinoma cell growth, metastasis, and angiogenesis [25,26]. VEGFA was upregualted in patients with endometrial cancer [27], and functions as a proangiogenic factor in endometrial carcinoma [28]. Therefore, miR-34b might contribute to angiogenesis and progression of endometrial cancer through upregulation of VEGFA, which needs to be further investigated.

ZFAS1 has been shown to promote colorectal cancer progression through competitively binding to miR-150-5p and upregulation of VEGFA [29]. Our results also showed that ZFAS1 enhanced expression of VEGFA in endometrial carcinoma cells through decrease in miR-34b. Therefore, the suppressive effects of VEGFA on endometrial carcinoma cell growth and metastasis might be caused by miR-34b-mediated decrease in VEGFA. Since anti-VEGFA, bevacizumab, was widely used for the prevention of various cancers [30], silence of ZFAS1 might be a promising therapeutic strategy for the prevention of endometrial carcinoma through inhibition of VEGFA.

In summary, lncRNA ZFAS1 was found to be enhanced in endometrial carcinoma tissues and cells. ZFAS1 functioned as a miR-34b sponge to promote the cell growth and metastasis of endometrial carcinoma through upregulation of VEGFA. However, the *in vivo* regulatory role of ZFAS1 on endometrial carcinoma tumor growth should be investigated to provide more information about the suppressive effect of ZFAS1 on endometrial carcinoma. Results in this study would give a new insight into the mechanism of progression of endometrial carcinoma and so as to suggest that lncRNA ZFAS1 might serve as a potential therapeutic target for the treatment of endometrial carcinoma.
